# Septic Thrombophlebitis of the Portal Vein: A Case Report

**DOI:** 10.7759/cureus.103559

**Published:** 2026-02-13

**Authors:** Megana Ballal, Eyong J Ly

**Affiliations:** 1 Hospital Medicine, University of California Los Angeles, Los Angeles, USA; 2 Internal Medicine-Pediatrics, University of California Los Angeles, Los Angeles, USA

**Keywords:** hypercoagulability, intra-abdominal infection, pylephlebitis, septic thrombophlebitis of portal vein, therapeutic anticoagulation

## Abstract

Pylephlebitis, a septic thrombophlebitis of the portal vein, typically arises as a complication of intra-abdominal infection. We report the case of a 53-year-old woman whose presenting symptoms included fever and abdominal pain. Imaging revealed portal vein thrombosis. An evaluation was performed to identify a potential underlying infectious source. She was treated with antibiotics and anticoagulation. This case highlights the diagnostic and therapeutic complexities in the evaluation and management of pylephlebitis.

## Introduction

Pylephlebitis is considered a type of portal vein thrombosis (PVT). It is a rare but potentially life-threatening complication of an intra-abdominal infection in an area drained by the portal venous system. Diverticulitis and pancreatitis are two intra-abdominal conditions commonly associated with pylephlebitis [1]. Its clinical presentation can be nonspecific, including fever and abdominal pain. Contrast-enhanced imaging has become critical for prompt diagnosis. The cornerstones of treatment are broad-spectrum antibiotics, with the regimen tailored to the source of infection and culture results, in combination with anticoagulation. While historically more controversial, anticoagulation is now increasingly supported, given evidence of improved outcomes [1]. PVT is uncommon in patients without cirrhosis. In patients who develop PVT in the absence of cirrhosis or another provoking factor, evaluation for an underlying thrombophilic condition is recommended [2]. Although advances in recognition and treatment have reduced morbidity and mortality in recent decades, maintaining a high index of clinical suspicion remains crucial for timely diagnosis.

## Case presentation

A 53-year-old woman with a history of eczema presented to the emergency department with fever, chills, epigastric pain, nausea, and vomiting. She had similar symptoms three weeks ago, which resolved after a week. Her symptoms recurred four days before presentation. Her temperature was up to 103 °F at home, for which she was taking Tylenol and ibuprofen one to two times a day. She tested negative for COVID, influenza, and respiratory syncytial virus one day earlier in the clinic. She had no prior surgeries and took no medications. She had a family history of Crohn’s disease. She had no history of tobacco, illicit drug, or significant alcohol use. 

On presentation, the patient was afebrile, and vitals were unremarkable with the exception of an elevated heart rate at 117 beats per minute. Mild epigastric tenderness to palpation was noted on physical exam. Initial labs are shown in Table 1. 

**Table 1:** Laboratory results. INR, international normalized ratio; AST, aspartate aminotransferase; ALT, alanine aminotransferase

Lab	Patient's lab value	Reference range and unit
White blood cell count	10.41	4.16-9.95 x 10E3/µL
Hemoglobin	12.8	11.6-15.2 g/dL
AST	134	13-62 U/L
ALT	218	8-70 U/L
Conjugated bilirubin	0.4	<=0.3 mg/dL
Total bilirubin	0.8	0.1-1.2 mg/dL
Total protein	8.9	6.1-8.2 g/dL
INR	1.3	0.8-1.2
Albumin	3.9	3.9-5.0 g/dL
C-reactive protein	27.8	<0.8 mg/dL

**Figure 1:**
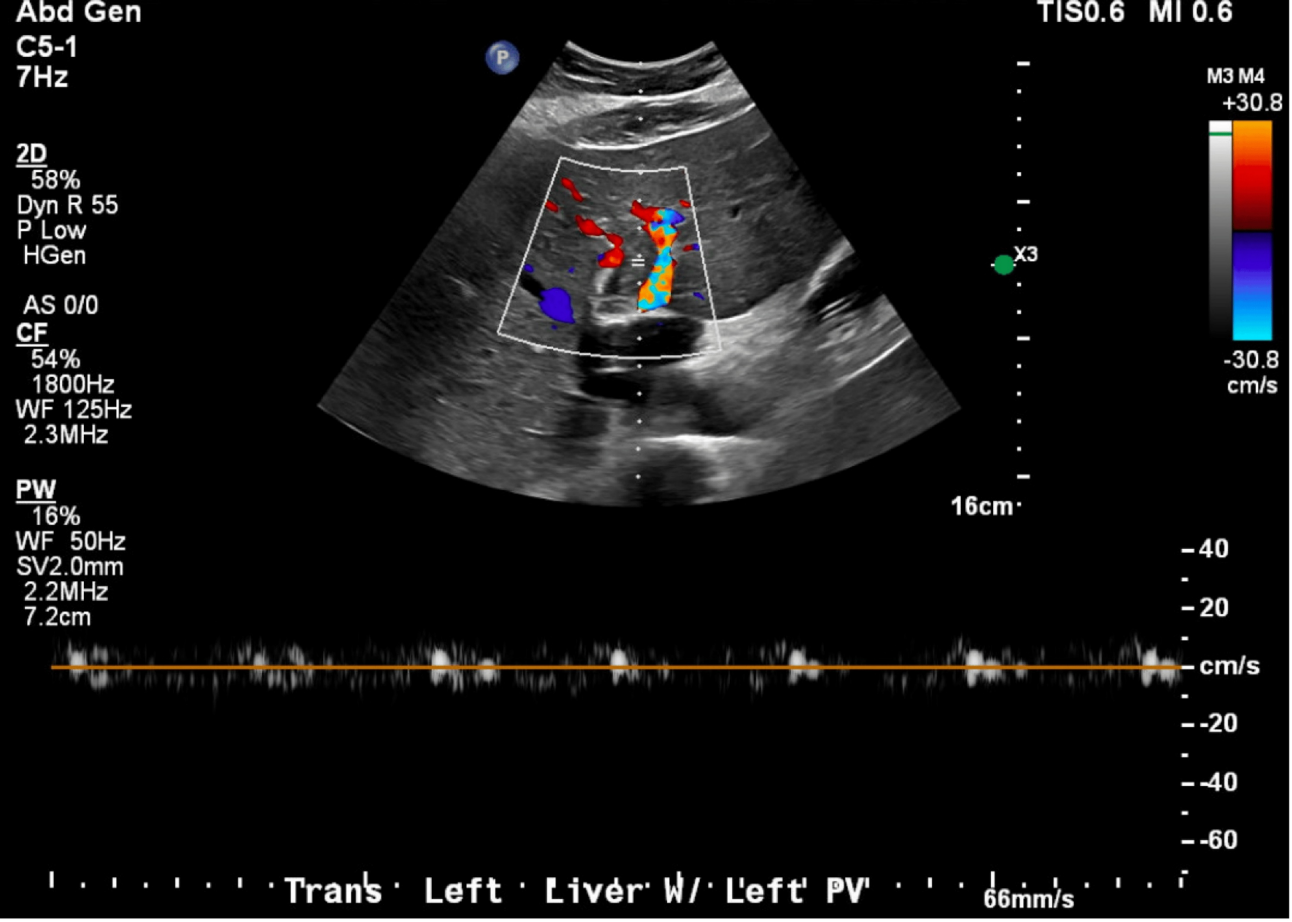
Abdominal ultrasound of the left portal vein. Ultrasound demonstrated a patent right portal vein with normal flow direction, as well as thrombosis of the left portal vein without an associated mass lesion.

An abdominal ultrasound showed an isolated left PVT (Figure 1).

**Figure 2:**
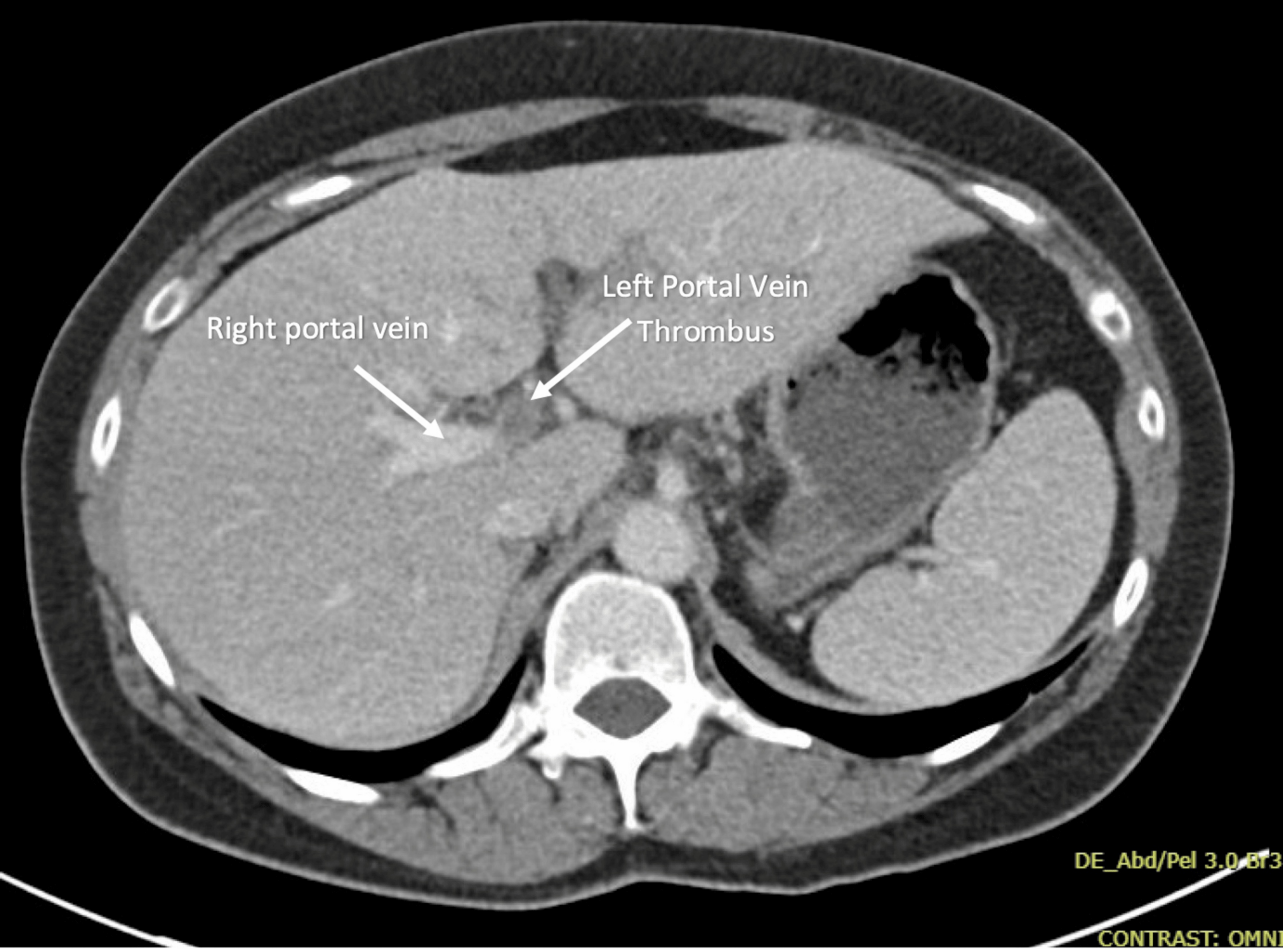
CT abdomen and pelvis with contrast showing a portal vein thrombus. An axial (transverse) CT image shows a filling defect in the left portal vein, consistent with complete thrombosis of the left portal venous system.

Blood cultures were drawn on admission, which were ultimately negative. On hospital day 2, repeat blood cultures were ordered given fever and malaise, and she was empirically started on ceftriaxone and metronidazole. An infectious disease specialist was consulted, and the patient was continued on these antibiotics based on the suspicion of an initial gastrointestinal infection with bacteremia that progressed to pylephlebitis. She was also started on a therapeutic dose of enoxaparin. A computed tomography (CT) scan of the abdomen and pelvis with contrast showed complete thrombosis of the left portal venous system with no specific findings of hepatic or enteric inflammation or malignancy (Figure 2).

**Figure 3:**
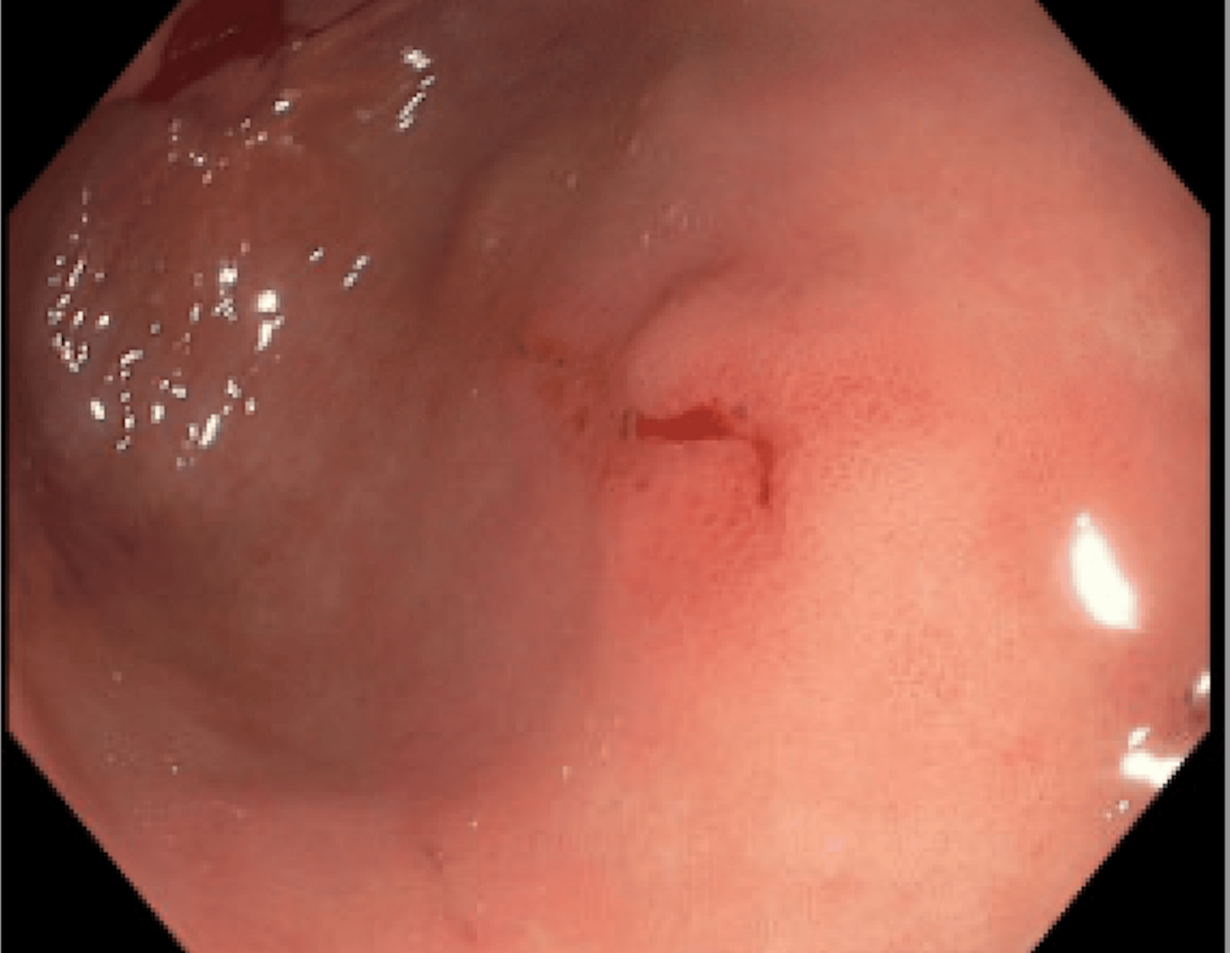
Antral ulcer.

One of the blood cultures was positive for Bacteroides fragilis. Urine culture was negative. She underwent endoscopy and colonoscopy on hospital day 5 as the source of bacteremia remained unclear. The endoscopy showed two clean-based antral ulcers, with a biopsy negative for *Helicobacter pylori* or intestinal metaplasia (Figure 3).

**Figure 4:**
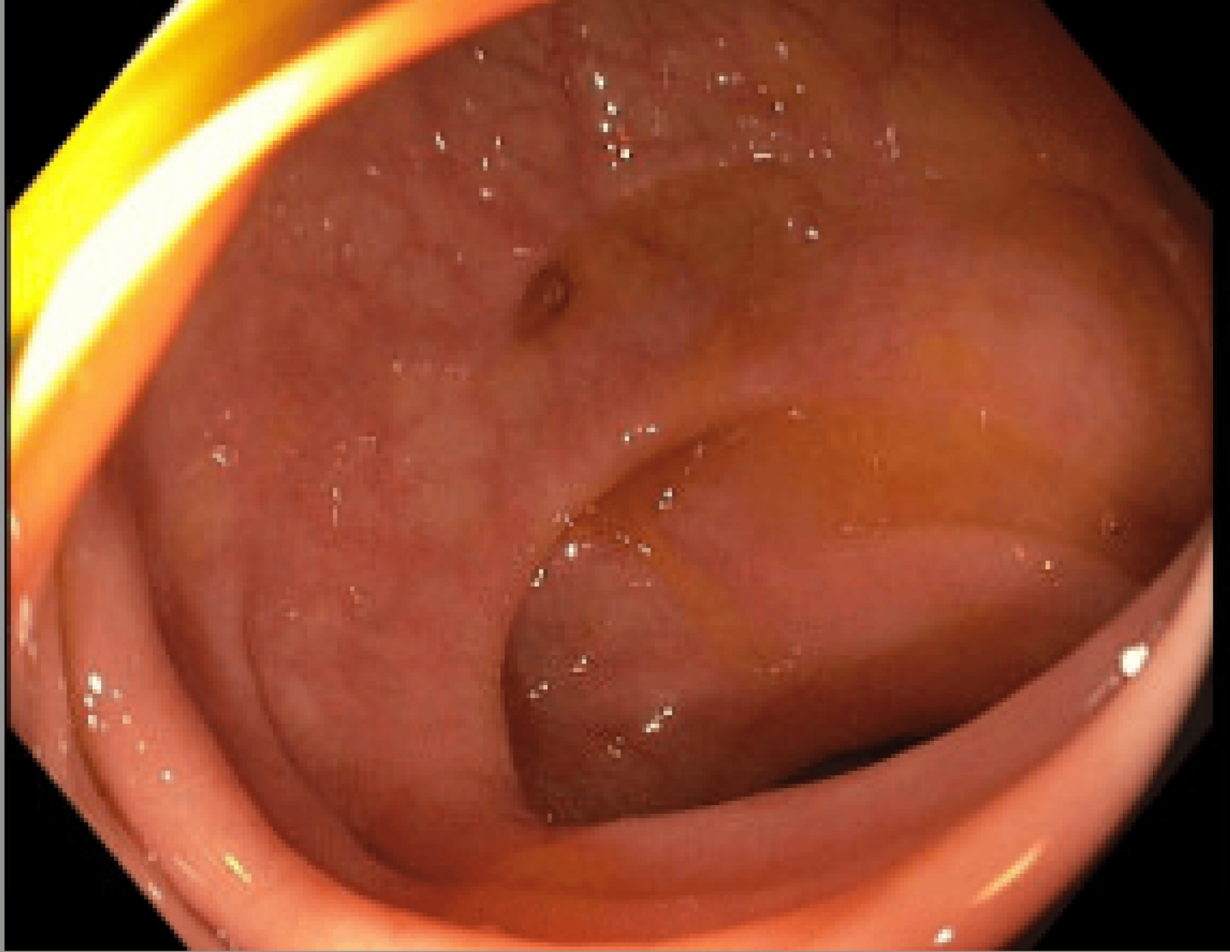
Colonoscopy demonstrating diverticulosis.

The colonoscopy demonstrated diverticulosis (Figure 4).

Evaluation for infectious or autoimmune causes of hepatitis was unremarkable. 

Given the resolution of fevers with clinical improvement and no sign of active infection on colonoscopy, ceftriaxone was discontinued after five days. Blood culture susceptibility testing demonstrated sensitivity to metronidazole. The patient was discharged on metronidazole with a plan to complete four weeks of treatment. She was transitioned to apixaban on discharge and also provided with pantoprazole twice daily, given the endoscopy findings. The patient was evaluated by hematology as an inpatient and had an outpatient follow-up. A hypercoagulable workup was performed and was negative for antiphospholipid syndrome, paroxysmal nocturnal hemoglobinuria, myeloproliferative neoplasms, inherited thrombophilias, and protein C or S deficiency. Therefore, her presentation was suspected to be due to pylephlebitis. The source of bacteremia remained unclear, although there was a suspicion of diverticulitis despite negative imaging based on her initial presentation and that diverticulosis was found on colonoscopy. She was advised to continue anticoagulation for six months.

## Discussion

Pylephlebitis is defined as septic thrombophlebitis of the portal vein. It was described by Waller as early as 1846 [1]. According to the American Association for the Study of Liver Disease, PVT is considered a heterogeneous condition in terms of cause, presentation, prognosis, and management [2]. PVT is less commonly seen in patients without cirrhosis [3]. Pylephlebitis is considered a type of PVT. It is an infrequent complication of intra-abdominal infections [4]. Although this condition is rare, it is associated with significant morbidity and mortality. Prompt diagnosis and treatment are crucial to prevent long-term complications. 

While the prevalence of PVT increases with the severity of cirrhosis, PVT remains uncommon among the general population [5]. The reported cases of pylephlebitis have increased over time, but the incidence remains low with an estimated 0.37-2.7 cases per 100,000 person-years [6]. A systematic review of 220 cases of pylephlebitis between 1971 and 2022 demonstrated that 70.5% of patients were male, with a median age of 50 years [6]. Pylephlebitis can occur as a complication of infection in an area drained by the portal system, with diverticulitis, pancreatitis, and appendicitis among the most common causes [1]. The diagnostic criteria for pylephlebitis are not clearly established. Choudhry et al. defined pylephlebitis as PVT within 30 days of an intra-abdominal inflammatory process with or without bacteremia [1]. Other review articles have defined this condition as PVT in patients with fever and bacteremia, although it was acknowledged that negative blood cultures shouldn’t rule out the diagnosis [6].

The clinical presentation of pylephlebitis can be nonspecific, with possible symptoms including fever, malaise, abdominal pain, and nausea [1]. Physical exam may or may not include right upper quadrant tenderness. Leukocytosis as well as elevated serum transaminases, C-reactive protein, and erythrocyte sedimentation rate occurred in the majority of patients [6]. *Escherichia coli*, Bacteroides spp., and Streptococcus spp. were the most commonly isolated pathogens, although no bacteria were identified in 30% of cases [6]. Ultrasound can be used for screening when there is suspicion for portal vein pathology, however it is operator dependent. Therefore, contrast-enhanced CT or magnetic resonance imaging (MRI) is useful to confirm the diagnosis and evaluate the clot burden [7].

Treatment of pylephlebitis starts with broad-spectrum antibiotics, with the specific medication depending on the suspected source of infection. Options for initial antibiotics include Ceftriaxone 2 g daily plus Metronidazole 500 mg every 8 hours, as well as monotherapy with Piperacillin-tazobactam 4.5 g every 8 hours. The duration of treatment is typically at least four to six weeks after symptom onset [6]. The role of anticoagulation has not been as clear; the use of anticoagulants has increased in recent years to include the majority of patients, especially when there is thrombus progression on repeat imaging or ongoing fever on appropriate antibiotic therapy [6]. Anticoagulation can increase the rate of thrombus resolution and decrease the rate of complications associated with chronic portal hypertension [8]. There is also no set recommendation on the exact duration of anticoagulation, with some data suggesting three to six months if there is no other cause of thrombophilia [1]. Invasive therapeutic approaches such as thrombectomy are not routinely used, as they are not the first-line treatment. 

Mortality from pylephlebitis has decreased to less than 10% since 2010, suggesting that diagnosis and treatment of this condition may have improved over time [6]. Complications associated with pylephlebitis include cavernous portal vein transformation or persistent portal vein thrombosis causing portal hypertension, dissemination of infection, including liver abscesses, and intestinal ischemia [6]. A study of 67 patients found that there was a 58% rate of portal vein thrombosis resolution among those who took anticoagulation, compared to 21% among those who did not [8]. Pylephlebitis is a rare but serious condition. The prognosis can vary depending on the timing of diagnosis and initiation of treatment, as well as identification of complications.

## Conclusions

Pylephlebitis is an uncommon but potentially life-threatening condition that can occur as a complication of an intra-abdominal infection. As illustrated by this case, its variable and nonspecific presentation underscores the need for a high index of suspicion, given its associated morbidity and mortality. Prompt imaging is essential to identify PVT and guide management, with antibiotics and anticoagulation serving as the mainstays of treatment. Ongoing research aims to clarify guidelines on anticoagulation and develop strategies to improve early recognition and intervention.
